# Use of spatiotemporal analysis of laboratory submission data to identify potential outbreaks of new or emerging diseases in cattle in Great Britain

**DOI:** 10.1186/1746-6148-7-14

**Published:** 2011-03-19

**Authors:** Kieran Hyder, Alberto Vidal-Diez, Joanna Lawes, A Robin Sayers, Ailsa Milnes, Linda Hoinville, Alasdair JC Cook

**Affiliations:** 1 Centre for Epidemiology & Risk Analysis, Veterinary Laboratories Agency, New Haw, Addlestone, Surrey KT15 3NB, UK; 2 Veterinary Laboratories Agency, Langford House, Langford, Bristol BS40 5DX, UK; 3 Centre for Environment, Fisheries and Aquaculture Science, Pakefield Road, Lowestoft, Suffolk NR33 0HT, UK

## Abstract

**Background:**

New and emerging diseases of livestock may impact animal welfare, trade and public health. Early detection of outbreaks can reduce the impact of these diseases by triggering control measures that limit the number of cases that occur. The aim of this study was to investigate whether prospective spatiotemporal methods could be used to identify outbreaks of new and emerging diseases in scanning surveillance data. SaTScan was used to identify clusters of unusually high levels of submissions where a diagnosis could not be reached (DNR) using different probability models and baselines. The clusters detected were subjected to a further selection process to reduce the number of false positives and a more detailed epidemiological analysis to ascertain whether they were likely to represent real outbreaks.

**Results:**

187,925 submissions of clinical material from cattle were made to the Regional Laboratory of the Veterinary Laboratories Agency (VLA) between 2002 and 2007, and the results were stored on the VLA FarmFile database. 16,925 of these were classified as DNRs and included in the analyses. Variation in the number and proportion of DNRs was found between syndromes and regions, so a spatiotemporal analysis for each DNR syndrome was done. Six clusters were identified using the Bernoulli model after applying selection criteria (e.g. size of cluster). The further epidemiological analysis revealed that one of the systemic clusters could plausibly have been due to Johne's disease. The remainder were either due to misclassification or not consistent with a single diagnosis.

**Conclusions:**

Our analyses have demonstrated that spatiotemporal methods can be used to detect clusters of new or emerging diseases, identify clusters of known diseases that may not have been diagnosed and identify misclassification in the data, and highlighted the impact of data quality on the ability to detect outbreaks. Spatiotemporal methods should be used alongside current temporal methods for analysis of scanning surveillance data. These statistical analyses should be followed by further investigation of possible outbreaks to determine whether cases have common features suggesting that these are likely to represent real outbreaks, or whether issues with the collection or processing of information have resulted in false positives.

## Background

Diseases of animals can have significant economic consequences through their direct (e.g. animal welfare, production, mortality, trade restrictions) and indirect effects (e.g. public health, rural access). The cost of foodborne zoonotic disease in the UK was estimated at £750 million in 2005 [1] including an estimated 70,000 cases of *Salmonella *each year [2]. New diseases have the greatest potential to have significant economic impact, for example, the cost of the UK BSE outbreak was estimated to exceed £2.3 billion and caused a reduction of 0.4% in GDP [1]. However, this impact could be reduced by early detection of outbreaks [3], as control measures could be implemented that limit the size of an outbreak (e.g. control of animal movements [4]). Routine national monitoring programmes are used to detect changes in animal disease status and contribute to the early detection of new and emerging diseases [5] and their importance is acknowledged in the Animal Health and Welfare strategy of the Department for Environment, Food and Rural Affairs (Defra). However, the data produced by these systems are noisy and difficult to interpret. Similar data issues are found in public health, so many statistical methods have been developed for the early detection of outbreaks and utilised in this field [6]. These methods have also been applied in the veterinary arena to assess changes in the incidence of existing diseases [7] and to look for new and emerging diseases [8].

Surveillance systems can be used to monitor the incidence of known diseases and conditions (e.g. *Salmonella*), and also to collect clinical information where a diagnosis cannot be reached. The collection of clinical information can contribute to scanning surveillance. Scanning surveillance has been defined as surveillance to monitor the health of defined populations in order to increase the likelihood that there will be timely detection of undefined or unexpected diseases, or of a change in the nature of an endemic disease. In Great Britain (GB), the FarmFile database has been used to collate epidemiological information on all clinical submissions received by the Veterinary Laboratories Agency (VLA) since 1998 and was developed to improve detection of new diseases (see [9] for a general review). Samples are submitted to VLA regional laboratories by veterinary practitioners for a variety of reasons, including investigation of clinical conditions on farm (diagnostic submissions) and further investigation of particular clinical conditions on farm (follow-up submissions). A submission may include samples from several animals. Submissions are grouped into broad syndromes based on the body system affected by the disease (e.g. enteric, musculoskeletal) and information about each submission is entered onto the FarmFile database. There are explicit case definitions that must be met if a diagnosis is to be entered. Some submissions may not yield a definitive diagnosis and these are termed diagnosis not reached (DNR). New or emerging diseases are unlikely to have a diagnosis code, so an outbreak of a new or emerging disease is likely to appear as an increase in the number of DNRs initially [9].

There are many statistical methods for early detection [6] that fit into two categories: prospective and retrospective methods. Prospective methods are real time methods, where analyses are repeated at regular time intervals using early detection systems to detect the next outbreak [3]. Retrospective methods are used to look for past outbreaks in a fixed data set [10].

Spatiotemporal methods are used routinely in veterinary epidemiology (e.g. [11-13]). These studies have focused on retrospective investigation of spatial clustering and associated risk factors. Betran *et al. *[14] used retrospective scan statistics to look for clusters in DNR data from animals showing nervous signs collected in England and Wales between 1999 and 2003. Prospective spatiotemporal methods have been used to detect disease outbreaks in the public health field both for specific diseases [15] and for syndromic data [16]. Kosmider *et al. *[8] used a Poisson regression model that accounted for seasonality and previous outbreaks to look for new outbreaks in DNR data. Here we have extended this temporal analysis of DNR data by using prospective spatiotemporal methods.

The aim of this study was to investigate whether prospective spatiotemporal scan statistics methods could be used to identify outbreaks of new and emerging diseases in scanning surveillance data. These methods were used to look for unusually high levels (numbers or proportions) of DNRs that could indicate an outbreak of a new or emerging disease, or an undetected increase of an endemic disease. Prospective methods were used to detect clusters that were subjected to further epidemiological analyses to determine if the clusters represented real outbreaks or aberrations in reporting. The conclusions of this study were used to make proposals about how scan statistics could be used in routine surveillance and to facilitate improvements in data collection.

## Methods

### Data extraction

Details of all submissions made to VLA regional laboratories from veterinary practitioners for investigation or follow up of clinical conditions in cattle were extracted from the FarmFile database (see [9]) for the period 1 January 2002 to 31 December 2007. These 187,925 records included information on the submission (e.g. date, reason, regional laboratory, diagnosis), holding (e.g. location, husbandry system) and animal (e.g. age, species, breed). The location of each holding was obtained as a Cartesian coordinate from a database of 577,836 holding locations and linked to the FarmFile data by county-parish-holding (CPH) number. These data were stored, manipulated and extracted using Microsoft Access 2003 (^© ^1992 - 2003, Microsoft Corporation).

### Selection of records for analysis

Submissions for which a diagnosis was reached were included in some of the analyses as denominators for DNR submissions to account for the fact that the number of submissions to FarmFile varied between locations (e.g. within a region) and over time (e.g. within and between years). This is consistent with the denominator data used in the routine analysis of DNR data [9,17,18]. Submissions in syndromes were selected for inclusion in these analysis, these were fetopathy, systemic disease, digestive disease, respiratory disease, musculoskeletal disease and nervous disease. A further reduction in the number of submissions included in the analyses resulted from the exclusion of submissions for which no presenting signs were recorded or representing a continuation of a previous submission. Submissions for which the testing carried out was not considered sufficient to reach a diagnosis were also excluded. This may occur for a number of reasons including a request to only test for a specific disease (e.g. *Salmonella*), resource limitations or sample quality issues.

A total of 70,175 submissions were used in the analysis including 16,925 DNRs and an additional 53,250 submissions for which a diagnosis was reached. The numbers of DNRs and denominators were aggregated each month and analyses were done on a monthly time step which is consistent with the routine analyses included in quarterly reports [17,18].

### Descriptive analysis

A descriptive analysis was done to look for trends, seasonality and potential covariates for DNRs, as all these factors have the potential to affect the detection of clusters. This was done both for numbers and proportions of DNRs (i.e. excluding and including denominators). Regional differences were assessed in order to justify using spatial methods and to look for trends. Seasonal patterns were investigated by calculating monthly averages. Differences between the number and proportion of DNRs submitted to each regional laboratory were assessed. Submissions can include samples taken from sick animals or in some cases entire carcasses. The effect of receiving a carcass on the probability of reaching a diagnosis was tested using chi-square tests, and an odds ratio was calculated (see [19] for general review). Statistics were done using Statistica 7 (^© ^StatSoft 1984-2006).

### Statistical analysis for detection of clusters of DNRs

Spatiotemporal scan statistics were used to detect a local excess of events and to test if this excess could have occurred by chance [10]. A scanning window was defined that was centred on a geographical location, and a likelihood ratio test was used to compare the number of cases within the scanning window to the expected number of cases in this window based on cases surrounding the scanning window. Many different scanning windows were tested and the window with the maximum likelihood is termed the most likely cluster. Free software has been developed called SaTScan (Kulldorff M. and Information Management Services, Inc. SaTScan (TM) software for the spatial and space-time scan statistics) to run both prospective and retrospective analyses for both spatial and temporal datasets. Various different probability models can be used [20], three of these were appropriate for our discrete data. Bernoulli models are used when there are cases and non-cases representing holdings with and without a disease [10]. Poisson models are used where the number of cases in each location follows a Poisson distribution and the number of cases is proportional to the population size [10]. Space-time permutation (STP) models require only case data and assume that the probability of a case being in an area given that it was observed in a particular time period is the same for all time periods [21].

Spatiotemporal scan statistics have been used previously to detect clusters of DNRs [14]. However, it is not clear how these methods should be applied to FarmFile (e.g. model, baseline, covariates) to detect a new or emerging disease or if these methods can be run routinely for all DNR syndromes. For this reason, a variety of prospective spatiotemporal early detection methods were used to investigate the effect on detection of DNR clusters of using the three different probability models (STP, Poisson, Bernoulli), baselines (fixed starting point, moving 2 year) and covariates (regional laboratory, carcass submissions). Trend was accounted for in the models and the maximum scanning window was set at 50% (see [20] for more information). Statistical significance was evaluated using Monte Carlo hypothesis testing [21]. All analyses were done using SaTScan 7.0.3 and data processing done using SAS 9.1.3 (^© ^2002 - 2003, SAS Institute).

**Figure 1:**
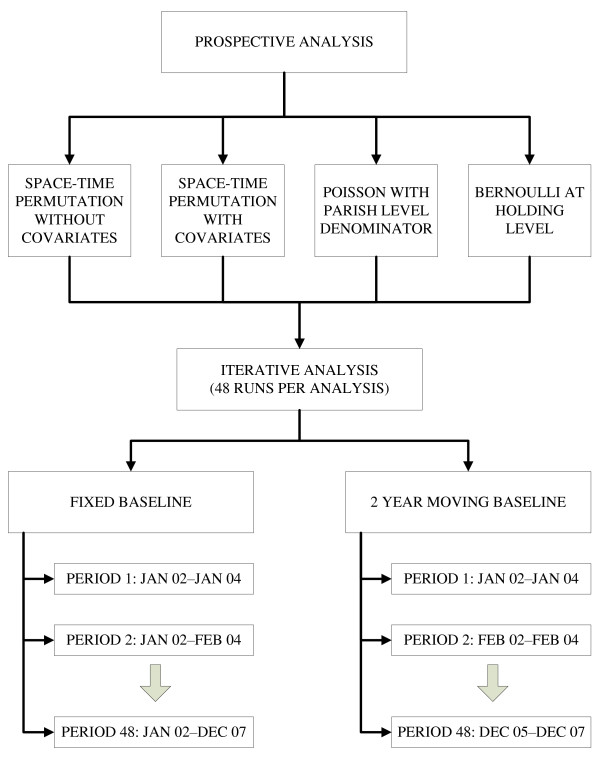
**Flow diagram showing analyses and runs done using SaTScan**.

The STP was run at parish level for each DNR syndrome, with and without regional laboratory and carcass as covariates and with a fixed and 24 month moving baseline (Figure 1). The Poisson model was run at a parish level, using the number of DNRs in a syndrome as numerator and all submissions in the syndrome as denominator for each baseline. The Bernoulli model was run at a farm level for each baseline, treating farms submitting a sample resulting in a DNR as cases and farms submitting a sample that was diagnosed as a control, farms with both case and control submissions were included as both case and control. The effect of covariates was only considered for STP, as inclusion of covariates in an analysis using denominator data led to data management problems, large file sizes and increased run times.

One analysis was done for each DNR syndrome, model, baseline and covariate. Each analysis comprised of 48 prospective SaTScan runs (one per month for the period January 2004 to December 2007) (Figure 1). The length of baseline increased by one month in each analysis for the fixed baseline, but remained constant at 2 years for the moving baseline (Figure 1). A moving baseline was used to account for changes in the processes that govern the submission of DNRs over time, as this can lead to a phase-shift in the baseline and make it more difficult to identify clusters. The fixed baseline analyses were adjusted for multiple testing [20] apart from in the Bernoulli model where it was not possible due to computational time.

### Selection of clusters most likely to represent outbreaks

Many clusters were detected in individual SaTScan runs, so selection criteria were used to identify the most promising clusters for each analysis that reduced the false positive rate. Initially the clusters that were ranked first and second most likely by SaTScan were selected for further analysis. Further selection of clusters was carried out by checking for geographical overlap using the radii and Euclidian distance between clusters detected in successive runs. The degree of overlap between the clusters was assessed by calculating the number of holdings that were in both clusters and the percentage of the total number of holdings in each cluster. We required 85% overlap between farms in clusters detected in two consecutive periods. Clusters that were present in three consecutive months were selected to reduce the chance of false positives and clusters with less than 300 holdings were selected, as larger clusters should be identified by other surveillance methods. Cut-off criteria for these measurements were defined and their sensitivity assessed.

### Epidemiological Investigation of clusters most likely to represent outbreaks

The clusters identified using the spatiotemporal methods described above may not represent real outbreaks of disease (an increase in the number of cases of a single disease). They may simply be unusually high random variations or aberrations in data recording. The absence of known outbreaks in the data set makes it difficult to assess the sensitivity and specificity of the early detection method, as the false detection rate cannot be calculated. Hence, clusters of DNRs detected using spatiotemporal methods were subjected to a further epidemiological investigation in order to determine if any clusters represented a real outbreak. Potential links between the submissions in each cluster were investigated by reviewing all of the data collected from each case at the time of submission (e.g. breed, farm type, herd size, age, test package, location, misclassification, presenting sign, case definition etc.). This was done to determine if the cases in the cluster were likely to be affected by the same disease, in which case it may represent a real outbreak.

## Results

### Descriptive analysis

**Figure 2:**
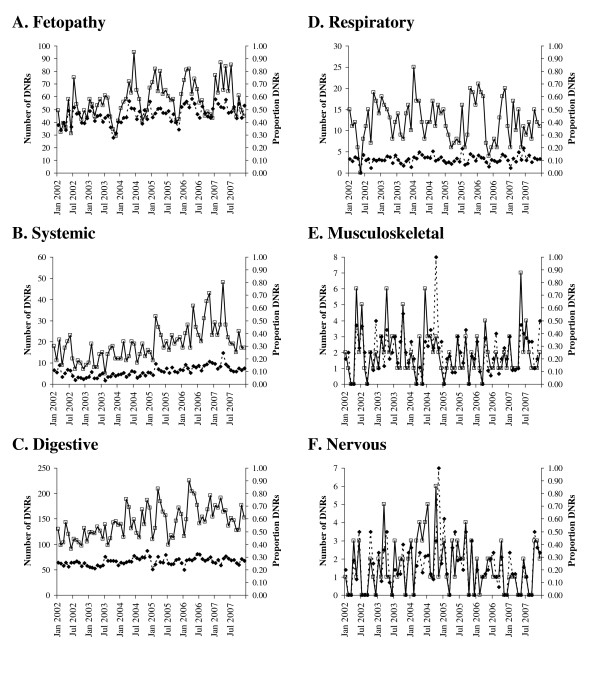
**Number and proportion of cattle submissions for which a diagnosis was not reached (DNRs) recorded on FarmFile between January 2002 and December 2007 for each syndrome**. A. Fetopathy; B. Systemic disease; C. Digestive disease; D. Respiratory disease; E. Musculoskeletal disease; and F. Nervous disease. Hollow squares, solid lines - number of DNRs; solid diamonds, dashed lines - proportion of DNRs.

**Figure 3:**
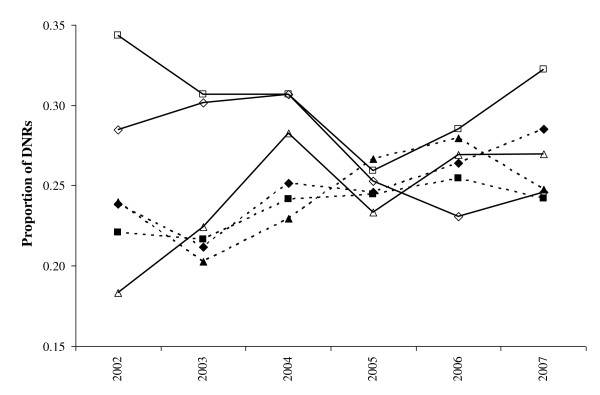
**Proportion of submissions in all syndromes used in these analyses for which a diagnosis was not reached (DNRs) by region**. Hollow squares, solid lines - southeast; hollow triangles, solid lines - southwest; hollow diamonds, solid lines - east; solid squares, dashed lines - mid & western; solid triangles, dashed lines - northern; and solid diamonds, dashed line - Wales.

There was variation in the number and proportion of DNRs found for each DNR syndrome with digestive and fetopathy having the largest number of DNRs (Table 1). Fetopathy had the highest proportion of DNRs, which was probably due to the difficulty in identifying the cause of an abortion [22]. There was temporal variation in the number and proportion of DNRs between syndromes (Figure 2). The number of systemic and digestive DNRs increased over time (Figures 2B&2C), as did the proportion of fetopathy and systemic DNRs (Figures 2A&2B). Musculoskeletal and nervous DNRs had the largest variation in the proportion of DNRs between time periods (Figures 2E&2F) due to the low numbers of submissions (Table 1). Variation in the proportion of DNRs in different regions decreased over time and the changes in proportion of DNRs over time varied among regions (Figure 3). This indicated that spatiotemporal methods should be used.

**Table 1:** Number of diagnosis not reached (DNR) submissions in each syndrome, proportion of total submissions in each submission that were DNRs and mean and range of the number and proportion of DNRs recorded by each Regional Laboratory (RL).

Syndrome	Total number of DNRs	Proportion of all submissions that were DNRs	Mean (range) of number of DNRs recorded in each RL	Mean (range) of proportion of DNRs recorded in each RL
Fetopathy	4111	0.46	257 (26, 817)	0.48 (0.35, 0.58)
Systemic disease	1359	0.10	85 (1, 325)	0.11 (0.00, 0.26)
Digestive disease	10322	0.27	645 (13, 1813)	0.26 (0.15, 0.32)
Respiratory disease	881	0.10	55 (1, 186)	0.10 (0.01, 0.18)
Musculoskeletal disease	138	0.24	9 (2, 31)	0.23 (0.00, 0.45)
Nervous disease	114	0.22	7 (1, 13)	0.23 (0.11, 0.46)
Total	16,925	0.24	1058 (57, 3173)	0.24 (0.17, 0.30)

Only includes DNRs that satisfy the selection criteria described in the data collation and selection section in the period 1 January 2002 to 31 December 2007.

Seasonal patterns in the number of DNRs were found for some DNR syndromes. For example, fetopathy was highest between January and July, digestive DNRs were lowest during the summer and respiratory DNRs rose in autumn. However, these patterns were no longer found when denominator data were included in the analysis. This suggested that it was important to include the denominator data in the analysis as the number of DNRs was proportional to the number of submissions for the syndrome.

Large differences in the numbers and proportions of DNRs recorded by each regional laboratory were found for all DNR syndromes. Significant differences between the diagnosis rates for carcass and non-carcass submissions were found for all DNR syndromes apart from nervous disease. There was a greater chance of reaching a diagnosis when a carcass was submitted for all syndromes (odds ratio > 1) apart from systemic disease where a diagnosis was less likely to be reached when a carcass was submitted (odds ratio < 1) (Table 2). These results indicated that both regional laboratory and carcass should be included as covariates.

**Table 2:** Number of submissions and odds of reaching a diagnosis for submissions with and without an entire carcass (significant chi-square tests are in bold).

	Number of submission with diagnosis reached		Number of submissions for which a diagnosis was not reached				
Syndrome	Carcass	No Carcass	Carcass	No Carcass	Odds Ratio	Chi-Square	Probability
Fetopathy	303	3883	158	3953	1.95	49.81	**< 0.0001**
Systemic disease	3310	8865	453	906	0.75	25.26	**< 0.0001**
Digestive disease	3081	25487	329	9993	3.67	533.5	**< 0.0001**
Respiratory disease	3406	4071	113	771	5.86	343.6	**< 0.0001**
Musculoskeletal disease	177	265	33	105	2.13	9.353	**< 0.0001**
Nervous disease	235	167	74	40	0.76	1.540	0.2146
Total	10512	42738	1157	15768	3.35	1562	**< 0.0001**

### Detection of clusters of DNRs

**Figure 4:**
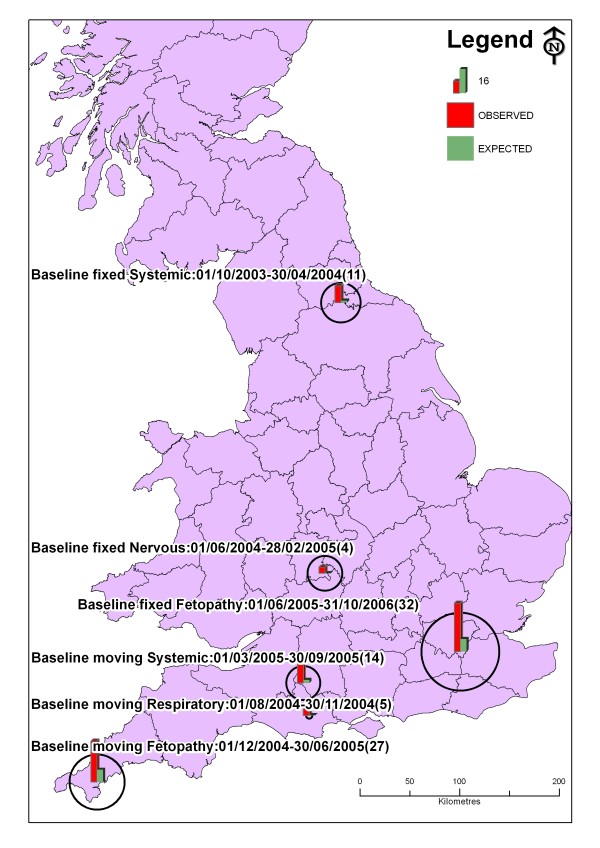
**Location and size of clusters of submissions for which a diagnosis was not reached (DNRs) detected using SaTScan using Bernoulli methods**. A. Fixed baseline and B. Moving baseline. Numbers in brackets indicate number of cases in cluster.

Processing times for each individual SaTScan run varied from minutes to a number of hours and the longest set of prospective runs took over one week (Bernoulli model with fixed baseline on digestive DNRs). It should be feasible to run routine individual analyses for quarterly reports [17,18] even though the complex analyses described here are computationally intensive. Many clusters were identified for all DNR syndromes using all methods. However, only 6 clusters of between 4 and 32 DNR cases were identified after the selection criteria were applied. All of these were identified using the Bernoulli model. Clusters were identified in the fetopathy and systemic syndromes using both fixed and moving baselines, in the respiratory syndrome using the moving baseline and in the nervous syndrome using a fixed baseline (Table 3). All clusters covered small areas and were found in the southwest, southeast, midlands and northeast of GB (Figure 4). Reducing the maximum size of clusters selected from 300 to 150 holdings had little effect on the number of clusters identified. However, decreasing the proportion of common holdings from 85 to 50% led to more clusters being identified.

**Table 3:** Characteristics of clusters of submissions for which a diagnosis was not reached (DNR) detected using Bernoulli models with DNR holdings as cases and all other submissions in a syndrome as controls.

Syndrome	Baseline	Start Date	End Date	Locations in cluster	Observed Cases	Expected Cases	Relative Risk	Probability
**Fetopathy**	Fixed	Jun 05	Oct 06	164	32	8.35	3.86	0.001
	Moving	Dec 04	Jun 05	176	27	8.11	3.37	0.009

**Systemic**	Fixed	Oct 03	Apr 04	144	11	1.29	8.74	0.011
	Moving	Mar 05	Sep 05	162	14	2.06	6.98	0.003

**Respiratory**	Moving	Aug 04	Nov 04	6	5	0.13	40.4	0.005

**Nervous**	Fixed	Jun 04	Feb 05	64	4	0.06	76.6	0.009

### Epidemiological investigation of clusters of DNRs

All of the clusters identified using SaTScan that satisfied the selection criteria were subjected to further epidemiological analysis using all of the information collected at the time of submission (Table 4). The nervous and respiratory clusters had very few cases making this analysis difficult. However, the respiratory cluster consisted of animals of different ages and presenting signs suggesting no single case definition, so was unlikely to represent an outbreak. No pattern was found for the fetopathy clusters due to misclassification of cases or in one of the systemic clusters (Table 4). As no single case definition was found for these clusters, they were unlikely to represent real outbreaks. If these methods were applied prospectively it is possible that collection of further information from affected farms may have revealed similarities or links between cases. The systemic cluster found with 12 cases between March and September 2005 was located on the border between Wiltshire, Dorset and Somerset. It included 8 holdings, the cases were mainly adults that exhibited signs of wasting and few animals were affected in each herd (1 to 5 animals in herds of 50 to 3000). Wasting in adults and low within herd prevalence are found in Johne's disease (*Mycobacterium avium subsp. *paratuberculosis). Further investigation showed that Johne's disease had been diagnosed on two of these holdings during the study period. Hence, this cluster could represent a local outbreak of Johne's disease.

**Table 4:** Number of holdings and outcome of epidemiological investigation of clusters to determine whether there were similarities between the cases included.

Syndrome	Start Date	End Date	Number of holdings	Similar presentation	Notes
**Fetopathy**	Jun 05	Oct 06	19	No	Mainly adult milking herds, but some misclassification
	Dec 04	Jun 05	19	No	Different breeds, herd size and some misclassification

**Systemic**	Oct 03	Apr 04	8	No	Diverse ages and presenting signs
	Mar 05	Sep 05	12	Yes	Adults with wasting could suggest Johne's Disease

**Respiratory**	Aug 04	Nov 04	3	No	Different age groups and presenting signs

**Nervous**	Jun 04	Feb 05	3	No	Insufficient cases

## Discussion

Methods used for the detection of outbreaks in animal populations have focused on the detection of known disease (e.g. [7]), have been used to look for previous outbreaks (e.g. [14]) or have been temporal in nature (e.g. [7]). Here we used prospective spatiotemporal methods to identify clusters of DNRs that could represent an outbreak of a new or emerging disease. Retrospective data were used to ensure that sufficient clusters were detected to allow the statistical methods to be evaluated. The selected methods would need to be implemented using prospective data to allow timely detection of outbreaks of emerging disease as described later in this discussion. These analyses detected a number of clusters and further epidemiological analyses indicated that one of these clusters might represent Johne's disease. All remaining clusters were either due to false positives or misclassification of submissions. Hence, prospective SaTScan analysis using DNR data can be used to identify: (1) new or emerging diseases; (2) outbreaks of existing diseases that may not have been diagnosed; and (3) highlight issues with existing data. We have focussed on identifying clusters of DNR submissions that may represent an outbreak of a novel disease although not all DNR submissions will represent cases of new diseases. There are many reasons why it may not be possible to reach a diagnosis for a particular submission including sampling animals at an inappropriate stage of disease, following therapeutic treatments or obtaining inconclusive test results. Submissions for which only a limited amount of testing was possible were excluded from these analyses, but it is still possible that DNR submissions subjected to a reasonable level of testing may not represent cases of a new disease. Investigation of clinical diagnosis decision support systems is necessary to determine whether it is possible to reduce the number of submissions for which a diagnosis is not reached. The occurrence of a new disease could result in changes in the total number of submissions to FarmFile but focussing on DNRs is thought likely to enhance the sensitivity of detection. The use of alternative sources of scanning surveillance data (e.g. clinical data from veterinary practices) to enhance outbreak detection should also be investigated further.

Seasonal changes in the numbers of DNRs were found for a number of the DNR syndromes (e.g. fetopathy, digestive) that can be explained by climate and associated farming practices (e.g. timing of calving, putting cows out to pasture). Inclusion of denominator data dampened these patterns, indicating that similar seasonal patterns were found in the numbers of submissions. This showed that use of the number of submissions for each syndrome was a reasonable denominator for this analysis. An alternative approach is to use the total number of farms in the population as a denominator, but this would not account for variation in submission rates over time. Using a submission based denominator helps to adjust for some of the biases associated with this voluntary reporting system. The appropriate choice of denominators for use within early detection systems using the FarmFile data is an area that needs further investigation.

FarmFile is a large database and a number of changes to the types of data and way in which it is entered have been made since its conception in 1998. There are problems with incomplete fields and data quality especially within the early years of the database. For example, about one third of all records used for this analysis did not include the herd size (making this difficult to include as a covariate) and some fetopathy DNRs have been misclassified as diagnosis reached in a different database field. These inconsistencies have been dealt with and are rare in new submissions, but do impact on any analyses that include historical data. The incomplete data fields have been addressed by making veterinary practitioners more aware of the reason behind the need for data collection (i.e. endemic surveillance and horizon scanning).

Another data quality issue was raised by the submissions that could not be included in these analyses because only limited testing was carried out. For example, the private veterinary surgeon wishes to rule out a specific disease, such as *Salmonella*, and therefore only requests this test. It is possible that a diagnosis could have been reached for these submissions if further testing was conducted. Including these potentially misclassified submissions as DNRs could reduce the probability of detecting a real outbreak of new disease. These analyses have contributed to the effort to improve data quality and enhance their value to stakeholders for effective surveillance. Defra subsidise the collection of these data and need to ensure that their value is optimised. This has led to the promotion of these issues by VLA and has resulted in a huge improvement in the quality of the surveillance data provided by veterinary practitioners in recent years.

Regional laboratory and the submission of a carcass were shown to be important in reaching a diagnosis. There was large variation in the proportion of DNRs found at different regional laboratories, which could be partly explained by differences between individual veterinary surgeons in defining a DNR. However, over the last few years significant efforts have been made to standardise recording of DNRs across veterinary surgeons. Generally, it was easier to reach a diagnosis if the whole carcass was submitted. This is not surprising as a more thorough examination is possible with a carcass and more tests can be done. However, for systemic submissions there was a significantly lower probability of reaching a diagnosis with a carcass submission. This may be due to the inclusion of animals affected with metabolic diseases in this syndrome for which tests need to be conducted in live animals or shortly after death.

A large window size was used in all models to ensure that widely distributed clusters could be detected. Cluster selection criteria were used to increase the specificity of detection. Clusters were detected using all models, but only clusters found using the Bernoulli model satisfied the selection criteria for further epidemiological analysis. This is possibly due to the fact that the Bernoulli analysis was done at a farm level, leading to more scanning windows being tested. Despite the conservatism of the cluster selection, there was still a large proportion of false positives (3/3 for the fixed baseline and 2/3 for the moving baseline) and there did not appear to be any reason for this clustering of disease in these farms. It is difficult to assess the sensitivity and specificity of these methods because we do not have data on the occurrence of actual outbreaks. Work is in progress to simulate data sets that represent different types of epidemics (e.g. fast local epidemic). This will be used to assess the sensitivity and specificity of SaTScan methods for detecting outbreaks using the data that would be collected in our surveillance database and allow final recommendations about the selection of appropriate methods.

Prospective methods can be run reasonably quickly (less than 10 hours), so could be run routinely alongside current temporal methods [8] and included in the quarterly disease trend reports [18]. However, as these methods might produce a number of false positives, a cascade of further investigation would be necessary to deal with each cluster. Initially, clusters should be discounted in a similar way to analysis in this study through investigation of the nature of the cluster (size, rank) followed by an epidemiological analysis. If the cluster is not discounted, then further herd level testing could be done to rule out existing non-notifiable diseases (e.g. bulk milk testing for Johne's disease). Finally, veterinary practitioners submitting samples should be approached for more information in order to identify a case definition and compare this to known exotic diseases.

Scanning surveillance is an important frontline tool in the detection of new and emerging diseases. It is founded upon a high level of expertise in veterinary pathology, to identify known conditions and characterise previously unknown or newly emerging conditions. Whilst a single new definitive diagnosis is sufficient to identify a new condition or emerging disease (e.g. Bluetongue), many conditions may not have pathognomonic signs and may be much more difficult to discern. In these circumstances, early detection methods can alert Veterinary Investigation Officers and others to the presence of an aberration in expected data and a review of the cases that appear to represent a cluster can be conducted. If such an approach is to be valuable, then it should have a high sensitivity and a relatively low specificity, so that more false positive results are found. These false positive clusters may be explained by other factors such as changes in submission decisions, testing or reporting. Otherwise, the probability is that a new or emerging disease would not be observed via an early detection analysis until the occurrence was already relatively great.

## Conclusions

The detection of a possible undiagnosed cluster of Johne's disease cases and clusters resulting from misclassification of data suggest that spatiotemporal methods can be used to identify clusters of undiagnosed known disease and to identify problems with misclassification of data. These methods may also have the potential to detect clusters of new or emerging disease. However, not all clusters detected using statistical analysis will represent real outbreaks and further epidemiological investigation is required to determine whether these statistical clusters represent real outbreaks. The ability of these epidemiological investigations to identify similarities between cases may be enhanced if theses analyses are done prospectively as further data collection and field investigations could be carried out. Spatiotemporal methods could be used alongside current temporal methods for analysis of scanning surveillance data. The Bernoulli method detected the most clusters, but a large proportion of these were false positives. Simulated data would provide a means of comparing the efficiency of different models within SaTScan and optimising sensitivity and specificity of detection for different classes of disease. A cascade of investigations is proposed that uses epidemiological analysis, farm level tests and discussions with veterinary practitioners once a cluster has been identified.

## Authors' contributions

KH led the work, provided direction for the analysis and wrote the paper. KH and JL did the descriptive analysis, AVD did the SaTScan analyses and AM did the epidemiological investigation of clusters. ARS, LH and AJC provided input into the direction of the analyses. All authors critically revised the manuscript and read and approved the final version.
